# Dissecting the Binding Interactions of the Chromatin Remodeler SMARCA4 with G‑Quadruplex DNA

**DOI:** 10.1021/acs.biochem.5c00750

**Published:** 2026-02-27

**Authors:** Sarah K. Madden, David Tannahill, Shankar Balasubramanian

**Affiliations:** † Yusuf Hamied Department of Chemistry, 2152 University of Cambridge, Cambridge CB2 1EW, U.K.; ‡ Cancer Research UK Cambridge Institute, Cambridge CB2 0RE, U.K.; § School of Clinical Medicine, University of Cambridge, Cambridge CB2 0SP, U.K.

## Abstract

DNA G-quadruplexes (G4s) are key structural features in chromatin that are important to genome function. G4s have an apparent capacity to recruit a wide variety of proteins, including chromatin remodelers, yet the molecular basis and biophysical principles governing these interactions remain poorly understood. Here, we sought to build insights into the interactions of chromatin remodeler SMARCA4 with G4s using a biophysical approach. We found that SMARCA4 selectively recognizes the G4 structure over duplex and single-stranded DNA. SMARCA4 binds a wide range of G4s with different topologies and loop lengths with similar low nanomolar affinities. SMARCA4 was also observed to have a longer residency time on the G4 structure compared to that of other known protein–DNA interactions. We also found that the D1 (DExx-c) helicase domain of SMARCA4, which is important for tethering SMARCA4 to chromatinized DNA, was the predominant binding domain for G4 recognition. Our findings reveal new insights into how G4s interact with proteins, which may have important implications for understanding G4-mediated genome mechanisms.

## Graphical abstract

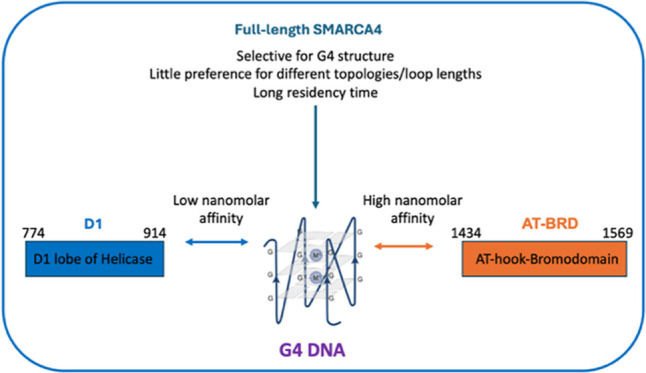

## Introduction

DNA G-quadruplexes (G4) are noncanonical four-stranded structures formed from stacked guanine tetrads stabilized by Hoogsteen bonds and coordination of monovalent cations positioned between the tetrads (Figure 1). G4s are key chromatin features that regulate diverse cellular processes such as transcription, epigenetic states, and genome stability.^1–3^ G4s can function as protein binding hubs to recruit a wide range of chromatin interacting proteins including transcription factors, such as SP1, and chromatin remodeling components including SMARCA4.^4–7^

**Figure 1:**
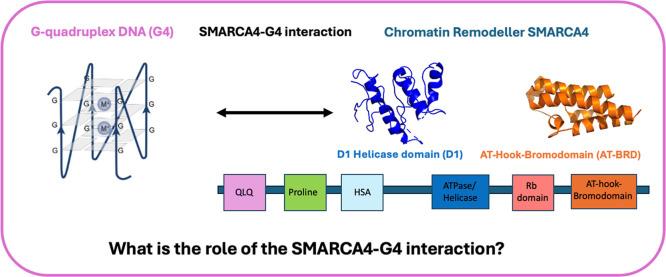
SMARCA4 has several key regions, including the AT-hook and bromodomain (orange, PDB ID 2GRC) and ATP/helicase domain (blue, PDB ID 6LTJ) as well as the QLQ (pink), proline (green), HSA (light blue), and Rb domain (coral).^15,25^ SMARCA4 interacts with G-quadruplex DNA (G4). We sought to understand the molecular basis and biophysics of this interaction and explored how SMARCA4 recognized the G4 through testing the binding of the D1 helicase domain (D1) and AT-hook-bromodomain (AT-BRD) to G4 DNA.

The molecular details by which G4s interact with proteins are still emerging.^8,9^ G4-binding interactions may be classified into three categories: loop-binding, groove-binding, and tetrad-binding.^10^ Although many proteins have been identified as G4-binding proteins, a detailed understanding of the molecular basis of these interactions is somewhat limited.^9–11^ A limited number of early studies with selected proteins have laid the groundwork for understanding G4–protein interactions at a molecular level. For example, a biophysical study by Williams et al. that found the protein SLIRP interacted with G4 DNA with nanomolar affinity via its RNA-binding RRM domain, and an X-ray crystal structure of the DHX36 helicase bound to a MYC G4 revealed that the N-terminal α-helical domain interacts with the top tetrad of the G4.^12,13^ There is a need to better understand how G4s interact with different protein classes of biological relevance.

An important class of G4-interacting proteins is chromatin remodelers, such as SMARCA4. SMARCA4 is the critical catalytic ATPase subunit of the SWI/SNF chromatin remodeling complex that drives chromatin remodeling through nucleosome ejection or repositioning.^14,15^ Mutations in SMARCA4 including the helicase domain are associated with a range of diseases including cancer.^16^ The interaction of chromatin remodelers, such as SMARCA4, have been identified through proteomic studies and further validated by ELISA and pull-down assays from nuclear extracts with synthetic G4 oligos.^17–20^ Further evidence for functional interactions between SMARCA4 and G4s comes from studies showing that SMARCA4 binding sites overlap with folded G4 features in the genome.^4^

SMARCA4 has been shown to have several domains with nucleic acid binding properties, including the helicase/ATPase domain, the AT-hook motif, and the bromodomain (Figure 1).^21–23^ The helicase domain is composed of two RecA-like domains, the DEAD-like helicase superfamily C-terminal domain (D1, DExx-c), and the helicase superfamily C-terminal domain (D2, HELIC-c) separated by an insertion and has been shown to be responsible for tethering SMARCA4 to chromatinized DNA.^21^ This binding interaction is inhibited by the long noncoding RNA myosin heavy-chain-associated RNA transcripts (Mhrt), which is also able to bind to the D1 helicase domain with a *K*_D_ in the high nanomolar range-low micromolar range.^21^

The AT-hook sits ten residues from the bromodomain toward the N-terminus of the protein, and an AT-hook and bromodomain composite (AT-BRD) was previously shown to bind double-stranded DNA in a multivalent manner with a low- to midmicromolar affinity.^22^ The bromodomain, usually known for binding to acetylated histones, with a preference for Histone 3 acetylated at Lysine 14 (H3K14Ac), has also been shown to bind ds DNA.^23–25^ The AT-hook has previously been shown to bind RNA.^26^ A fundamental understanding of how SMARCA4 is able to recognize G4 structures could help give insight into the functional role of this interaction. An appreciation of how this interaction relates to the binding preferences of naked duplex DNA and chromatinized DNA may further contribute to our understanding of its mechanistic significance.

## Materials and Methods

### Protein Expression and Purification

Helicase lobe D1 was cloned into the pMAL-c6T vector (residues 774–914) and expressed as an N-terminal MBP-fusion construct first reported by Han et al.^21^ in BL21 DE3 cells and grown in LB media at 37 °C until an O.D. of 0.5–0.6 was reached and induced with 0.5 mM IPTG and incubated overnight at 20 °C. Cells were pelleted at 5000 rpm for 5 min and resuspended in 35 mL of 50 mM *Tris* pH 7.4, 500 mM NaCl, 5% glycerol, 1 mM DTT, and SigmaFAST protease inhibitor tablet (EDTA-free) lysed using a cell homogenizer. The lysed cells were pelleted at 17 000 rpm for 45 min, and the cleared lysates were incubated with 4 mL Ni-NTA beads for 1 h at 4 °C. The beads were then washed using 3 × 50 mL washes with 50 mM *Tris* pH 7.4, 500 mM NaCl, 30 mM Imidazole, 5% glycerol, and 1 mM DTT and eluted with 10 mL 50 mM *Tris* pH 7.4, 200 mM KCl, 300 mM Imidazole, 5% glycerol, and 1 mM DTT. The eluant was further purified using a 26/600 Superdex 75pg column via gel filtration chromatography in 50 mM *Tris* pH 7.4, 200 mM KCl, 5% glycerol, 1 mM DTT, and 1 mM MgCl_2_.

The AT-hook-Bromodomain (residues 1434–1569) was cloned into the pGEX-6P-1 plasmid and expressed as a N-terminal GST-fusion as previously reported by Morrison et al., which was later cleaved.^23^ BL21 DE3 cells were grown at 37 °C until an O.D. of 0.8 was reached and induced with 0.3 mM IPTG and incubated overnight at 20 °C. Cells were pelleted at 5000 rpm for 5 min and resuspended in 35 mL of 20 mM *Tris* pH 7.5, 1 M NaCl, 3 mM DTT, 0.5% Triton, 0.5 mg/mL lysozyme, and SigmaFAST protease inhibitor tablet (EDTA-free) lysed using a cell homogenizer. The lysed cells were pelleted at 17,000 rpm for 45 min, and the cleared lysates were incubated with 6 mL GST beads for 2 h at 4 °C. Cells were washed with 3 × 50 mL of 50 mM Potassium Phosphate pH 7. NaCl (1 mM) and the GST tag were cleaved overnight at 4 °C with 2 units of preScission protease/100 μg of target protein in 50 mM Potassium Phosphate pH 7.4, 50 mM KCl, 1 mM DTT, and 0.5 mM EDTA. The cleavage mixture was incubated again with GST resin in 50 mM Potassium Phosphate pH 7.4, 50 mM KCl, 1 mM DTT, and 0.5 mM EDTA to capture and remove the protease and any uncleaved protein for 2 h at 4 °C.

Full-length SMARCA4 (UniProt accession ID P51532) was purchased from AscentGene with an N-terminal His-tag in 20 mM *Tris*, 100 mM NaCl, 1 mM DTT, 0.5 mM EDTA pH 7.9, and 20% glycerol from expression in sf21 cells.

### Oligonucleotide Annealing

HPLC-purified oligonucleotides were purchased from Sigma-Aldrich. For G4s and the single-stranded mutants, DNA was annealed in 10 mM *Tris* and 100 mM KCl pH 7.4. For double-stranded DNA, the forward strand and its reverse complement were annealed in a 1:1 ratio in 10 mM *Tris* and 100 mM NaCl at pH 7.4. All oligonucleotides were heated at 95 °C for 5 min followed by gradual cooling to 20 °C.

### CD Spectroscopy

The oligonucleotide structure was analyzed using Circular Dichroism. 10 μM oligonucleotide was analyzed in 10 mM *Tris* and 100 mM KCl, pH 7.4, between 200 and 330 nm with 1 nm increments at a scan speed of 0.5/s per time point at 25 °C using an Applied Photophysics Chirascan spectrophotometer. Three readings were taken and averaged.

### Biolayer Interferometry

All biolayer interferometry binding experiments were performed on an OCTET RED96 instrument at 25 °C with 100 nm of Biotinylated oligonucleotide loaded onto streptavidin biosensors. Binding curves from the titrations were fitted with a 1:1 global fit with Savitzky–Golay filtering. For the binding assay with full-length SMARCA4, a baseline of 400 s, a loading step of 300 s, and association and dissociation of 1500 s were used in 25 mM *Tris*, 100 mM KCl, 20% glycerol, 2 mM EDTA, 1 mM DTT, and 2% BSA, pH 7.9. For the binding assay with AT-BRD, a baseline of 400 s, a loading step of 200 s and association and dissociation of 200 s of the AT-BRD titration were used in 50 mM Potassium Phosphate, 50 mM KCl, 1 mM DTT, 0.5 mM EDTA, and 0.1% BSA, pH 7. For the binding assay with MBP-D1, a baseline of 300 s, a loading step of 240 s, and association and dissociation of 750 s of the MBP-D1 titration was used in 50 mM *Tris*, 200 mM KCl, 5% glycerol, 1 mM DTT, 1 mM MgCl_2_, and 0.2% BSA, pH 7.

## Results and Discussion

### SMARCA4 Has Selective and High-Affinity Binding to Different G4 Topologies

We sought to build a molecular picture of the dynamics of the SMARCA4-G4 interaction and how it might perturb remodeling processes. We therefore set out to (1) elucidate the selectivity of the interaction for the G4 structure and the preference for different topologies, (2) investigate the residency times of the SMARCA4-G4, sequestering the protein from other binding sites, and (3) understand the molecular recognition of the SMARCA4–G4 interaction and whether this gives clues on how the interaction could affect protein function.^27^

First, we sought to examine the affinity of SMARCA4 for G4 structures and any preference for different G4 topologies. G4s can adopt a range of topologies due to different combinations of strand direction resulting in parallel, antiparallel, or hybrid structures. All such topologies may exist within the genome, though parallel G4s are thought to be the most common.^27^ We first investigated the interaction of SMARCA4 with genomic G4s that comprise different topologies or loop lengths. To explore the effect of topology, we selected a range of known and previously characterized G4-forming synthetic oligonucleotides that included three parallel G4s (Kit1 G4, Pu27 G4, and VEGF G4), an antiparallel G4, Kit* G4, and a hybrid G4, BCL2 G4 (Supporting Information Table S1 and Supporting Information Figure S1).^5,28–33^ We first confirmed that these sequences could indeed adopt a G4 structure in vitro using Circular Dichroism spectroscopy and that the resulting spectra were consistent with previously published literature (CD, Supporting Information Figure S1). We next used biolayer interferometry (BLI) to measure SMARCA4 binding to the folded G4s. All G4s tested were observed to bind recombinant full-length SMARCA4 with similar low nanomolar affinities, with a slight preference for parallel G4s (e.g., *K*_D_ of 7.39 ± 3.6 nM for KIT1 G4) over hybrid (e.g., *K*_D_ of 12.1 ± 3.2 nM for BCL2 G4) and antiparallel G4s (e.g., *K*_D_ of 17.6 ± 3.2 nM Kit* G4, Figure 2). Several other proteins have also been shown to bind G4s with low nanomolar affinities such as SP1 and nucleolin.^5,34,35^ BLI also provides kinetics measurements of binding, showing that the high affinity is driven by a slow off-rate (*k*_off_) of the order of 10^–4^ to 10^–5^ s^–1^ (i.e., a residency time of 10,000 s to 100,000 s). This off-rate is slow relative to most protein–nucleic acid interactions, where a broad range of off-rates are observed, and values less than 10^–3^ s^–1^ are relatively rare.^36,37^ For example, for the interaction of transcription factors with double-stranded DNA, off rates can vary from 10^–4^ s^–1^ to 0.1 s^–1^ or higher, such as the interaction of transcription factors such as NF-KB with DNA, which has a residency time of around 100 s^38^

**Figure 2:**
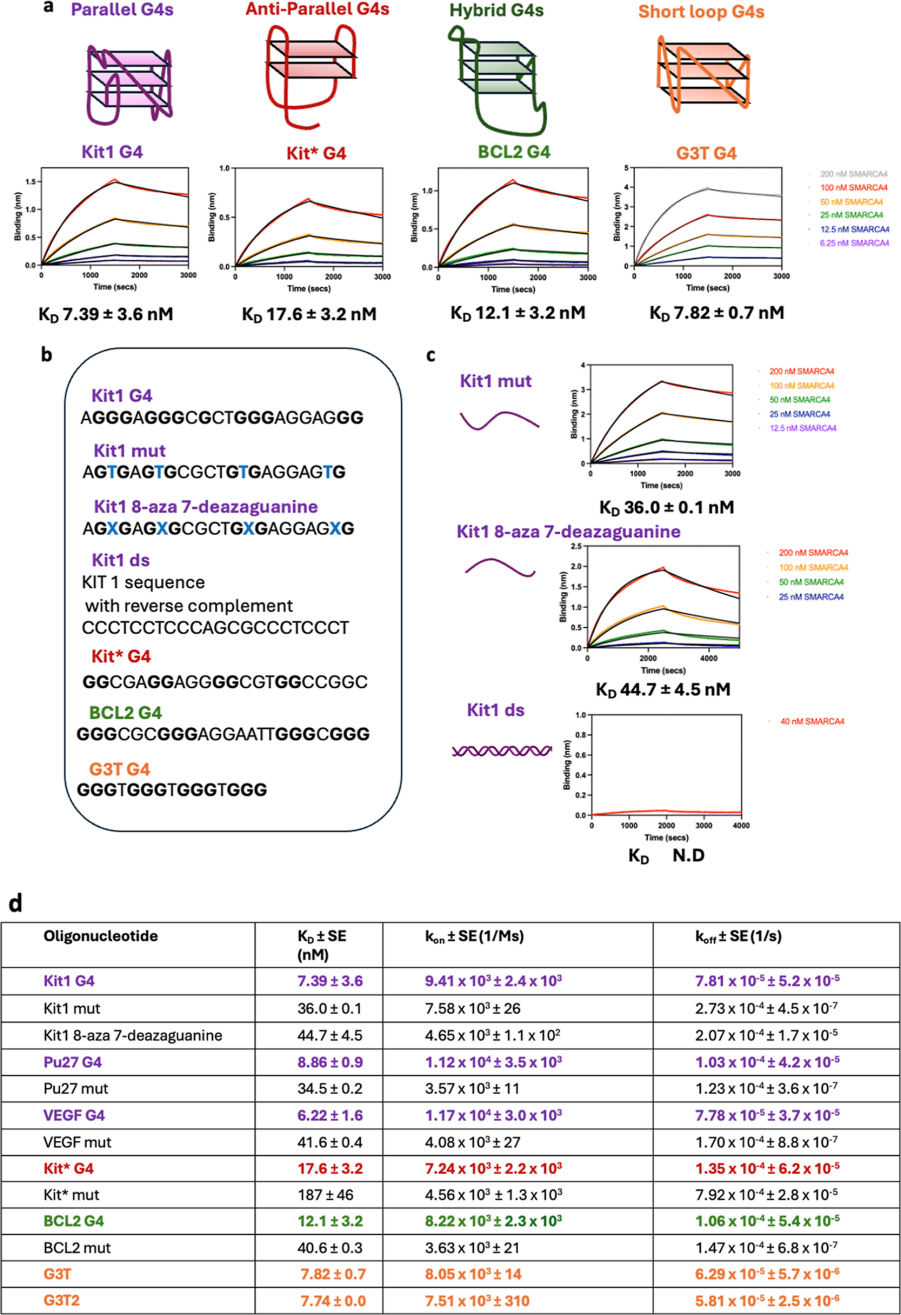
SMARCA4 binds tightly and selectively to G4s with different topologies and loop lengths. (A) Biolayer interferometry (BLI) with 100 nM immobilized oligonucleotide in 25 mM *Tris*, 100 mM KCl, 20% glycerol, 2 mM EDTA, 1 mM DTT, and 2% BSA, pH 7.9 shows SMARCA4 binds to parallel (purple, e.g., KIT1 G4), antiparallel (red, i.e., Kit* G4), hybrid (green, i.e., BCL2 G4), and short loop (orange, i.e., G3T G4) G4s with low nanomolar affinity. (B) Example oligonucleotide sequences used for BLI studies with tetrad forming guanines shown in bold and mutations disrupting tetrad formation shown in blue. All oligonucleotide sequences used in this study are provided in Supporting Information Table S1. (C) SMARCA4 shows selective binding for the G4 structure over ss and ds DNA (at least 3-fold greater affinity compared to control oligonucleotides with point mutations or 8-aza 7-deazaguanine substitutions that prevent G4 folding). (D) Summary table of all tested G4s: parallel (purple), antiparallel (red), hybrid (green), and short loop (orange) G4s and their corresponding control oligonucleotides that cannot form G4s.

To begin to understand whether G4s are preferential sites for SMARCA4 engagement in the genome, we next evaluated whether SMARCA4 shows selective binding for folded G4 DNA over single-stranded (ss) DNA controls that are unable to fold into a G4 structure. The ss DNA controls either substituted key guanines bases with thymine to prevent Hoogsteen bond formation and therefore G4 formation or substituted key guanines with the isomer 8-aza-7-deazaguanine substitutions to preclude Hoogsteen bonds and G4 formation.^39^ These ss DNA controls were unable to form the G4 structure in vitro, as shown by circular dichroism (Figure 2b, Supporting Information Figure S1, Supporting Information Table S1). For these ss DNA controls, BLI measurements showed that SMARCA4 selectivity binds G4 structures over ss DNA controls with a 3- to 10-fold preference for the G4 (e.g., *K*_D_ of 7.39 ± 3.6 nM for KIT1 G4 and *K*_D_ of 36.0 ± 0.1 nM for KIT1 mut and *K*_D_ of 44.7 ± 4.5 nM for KIT1 8-aza 7-deazaguanine ss controls, Figure 2c).

We next evaluated whether SMARCA4 was able to differentiate between the folded G4 structure and the double-stranded (ds) DNA control to further understand how SMARCA4 may discriminate over different structures. For the ds DNA control, we used the duplex form of the Kit1 G4 sequence (Figure 2c, Supporting Information Table S1). We observed only minimal binding to ds DNA and were unable to calculate an accurate *K*_D,_ which would be at least 10-fold weaker than that observed for the G4 structure (e.g., 7.39 ± 3.6 nM for KIT1 G4). Together with the results for ss DNA, these results identify G4s as key nucleic acid features of SMARCA4 interaction.

### Role of G4 Loops in SMARCA4 Binding

In the genome, G4 structures can have different interconnecting loop lengths between tetrads. To understand whether SMARCA4 differentiates G4s based on their loop lengths, we evaluated SMARCA4 binding to a range of G4s with varying loop lengths. The naturally occurring G4s have various lengths of loop, with the longest loop being three (Kit* G4), four (VEGF G4), five (Kit1 G4), six (Pu27 G4), and seven (BCL2 G4) bases. We also wanted to explore the effect of having only very short length loops. For this, we used previously reported synthetic G4 oligonucleotides with very short loops with one or two thymines in the loops (G3T and G3T2, Supporting Information Table S1, Figure 2).^40^ When comparing the binding of these synthetic G4 oligonucleotides and the naturally occurring G4s to SMARCA4, BLI measurements found that G3T and G3T2 have similar low nanomolar binding affinities for SMARCA4 (e.g., *K*_D_ of 7.82 ± 0.7 nM for G3T G4 and 7.74 ± 0.0 nM for G3T2 G4) to the other naturally occurring G4s with longer loops (e.g., *K*_D_ of 7.39 ± 3.6 nM for KIT1 G4). We also see no clear effect in the affinities of G4s and the length of loop among the naturally occurring G4s (e.g., for the parallel G4s *K*_D_ of 7.39 ± 3.6 nM for Kit1 G4, 8.86 ± 0.9 nM for Pu27 G4, and 6.22 ± 1.6 nM for VEGF G4). These results suggest that the loops of the G4 do not play a major role in driving SMARCA4 binding, in contrast with the G4-interacting protein Nucleolin, where the protein primarily recognizes the exposed loops and flanking regions of the G4, with little interaction observed with the tetrad or core.^35^ Instead, other binding modes may be at play such as that seen for DHX36, which interacts with the top of the tetrad or like the protozoan analogue of human POT1, which interacts with the groove region between loops.^13,41^ This result, coupled with minimal differences in binding affinities in SMARCA4 interactions with different G4 topologies, suggests that SMARCA4 is relatively nondiscriminatory for particular G4 features beyond the core G4 structure and that SMARCA4 is likely recognizing the G4 tetrad as the key signal of G4 presence independent of loop length or topology. Biologically, this would suggest that most folded G4s in the genome could be potential sites of SMARCA4 recruitment.

### SMARCA4 Binds to G4s via the Helicase Domain

We next considered which subdomain of SMARCA4 recognizes G4 DNA as this will influence how SMARCA4 remodels chromatin. SMARCA4 is a complex multidomain protein in which several domains are thought to interact with nucleic acids. We focused on the AT-hook with bromodomain and D1 lobe of the helicase domains as these have previously been shown to interact with nucleic acids with albeit micromolar affinities.^21–23^ Recombinant proteins for each of these domains were expressed and purified for G4 binding assays using BLI (Methods; Supporting Information, Figure 3). The AT-BRD bound G4s with micromolar affinity and showed fast on and fast off kinetics (e.g., *K*_D_ of 528 ± 94 nM for Kit1 G4). In marked contrast, the D1 helicase domain (expressed as a maltose binding protein fusion MBP-D1) bound G4s with nanomolar affinity with slow binding and slow dissociation kinetics (e.g., *K*_D_ of 14.1 ± 0.2 nM for KIT1 G4 with a *k*_on_ of 9.30 × 10^3^ ± 200 M^–1^s^–1^ and a *k*_off_ of 1.31 × 10^–4^ ± 1.1 × 10^–6^ s^–1^). As a control, the MBP protein alone showed no binding to G4s (Supporting Information Figure S2). These measurements identify the D1 helicase domain as the predominant driver of SMARCA4–G4 interactions although slightly weaker than that for full-length SMARCA4 (e.g., *K*_D_ of 7.39 ± 3.6 nM for full-length SMARCA4 binding to KIT1 G4 vs 14.1 ± 0.2 nM for MBP-D1 binding to KIT1 G4), suggesting other regions in SMARCA4 may also contribute to binding. Importantly, ss DNA control oligonucleotides that were unable to fold into G4s display higher *K*_D_ values for the D1 helicase domain compared to their corresponding G4s, indicating that this domain is responsible for the selectivity of SMARCA4 for G4s (e.g., 42.7 ± 1.2 nM for BCL2 G4 compared to 434 ± 1.4 nM for BCL2 mut ss DNA). Our findings that the helicase domain is a key contributor to G4 binding further emphasizes the importance of G4–protein interactions in chromatin, and it has previously been demonstrated that this domain is also responsible for tethering SMARCA4 to chromatin and a long noncoding RNA that bound to this region was shown to inhibit protein function.^21^

**Figure 3:**
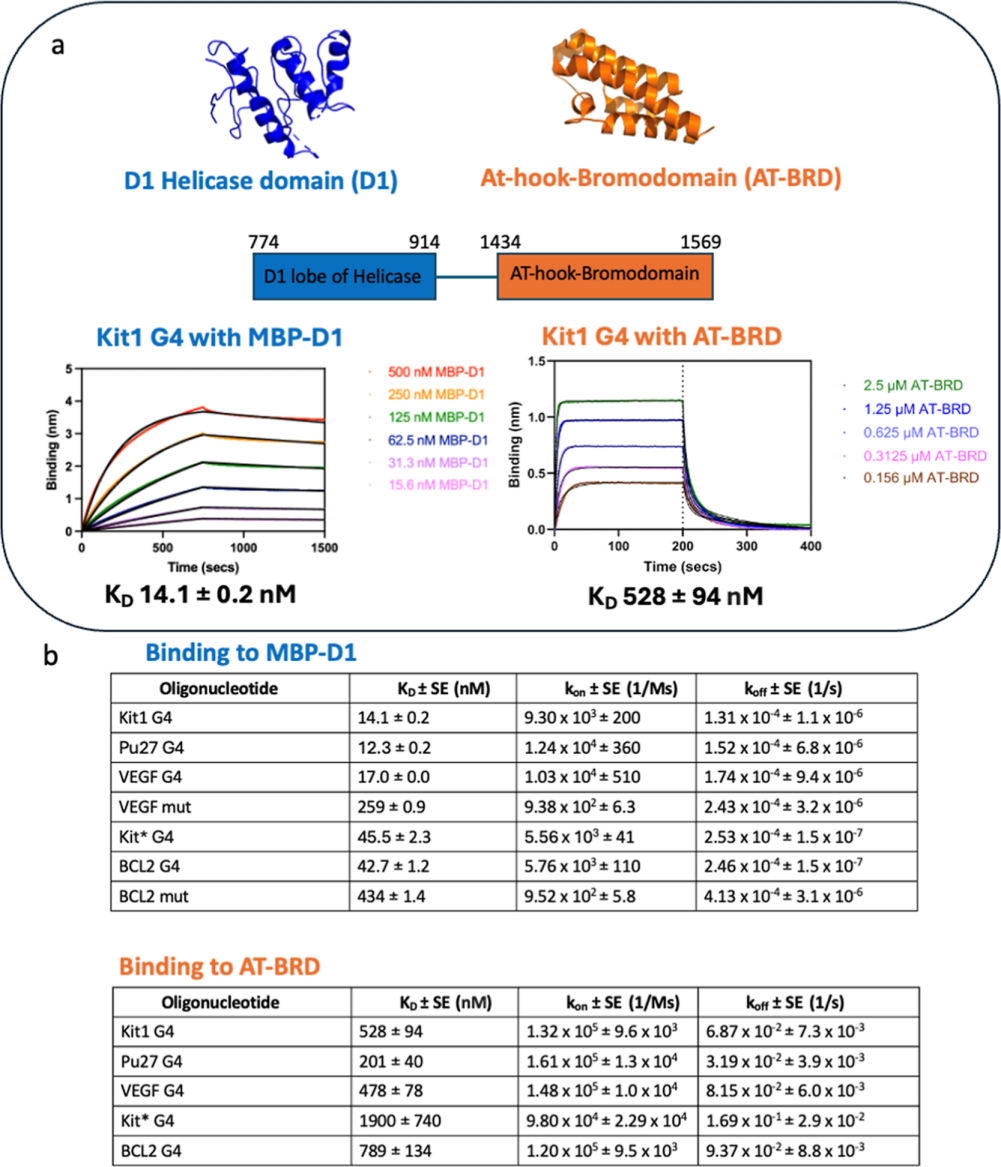
SMARCA4–G4 interaction is primarily mediated by the SMARCA4 D1 helicase domain. (A) To investigate which SMARCA4 domains contribute to G4 binding, different SMARCA4 domains known to interact with nucleic acids were tested for G4 binding by BLI: the D1 lobe of the helicase (blue, PDB ID 6LTJ) with 100 nM immobilized biotinylated oligonucleotide in 50 mM *Tris*, 200 mM KCl, 5% glycerol, 1 mM DTT, 1 mM MgCl_2_, and 0.2% BSA, pH 7, and the AT-hook-bromodomain with 100 nM immobilized oligonucleotide in 50 mM potassium phosphate, 50 mM KCl, 1 mM DTT, 0.5 mM EDTA, and 0.1% BSA, pH 7 (orange, PDB ID 2GRC).^15,25^ BLI revealed nanomolar affinity for G4s for a MBP-tagged D1 helicase domain (blue) and micromolar affinity for AT-BRD (orange). These results suggest that the D1 helicase domain predominantly drives SMARCA4 binding to G4s. (B) Table showing measured affinities and kinetics by BLI. MBP-D1 showing selectivity for the G4 structure over mutant oligonucleotides (Supporting Information Table S1) unable to form a G4 structure.

## Conclusion

Our aim was to gain a molecular picture of the SMARCA4–G4 interaction to better understand its importance and biological role. Through our biophysical studies, we found that SWI/SNF chromatin remodeler component SMARCA4 shows high selectivity for G4s, with high affinity for a range of different G4 structural types tested. Our findings emphasize G4s as key sites for SMARCA4 recruitment, in a similar manner to how SWI/SNF recruitment to different chromatin sites has been shown to be directed by the presence of different transcription factors and chromatin modifications.^42–45^ Of particular note was our observation that SMARCA4 has a relatively long residency time on the G4 structure owing to a relatively slow off-rate. Longer residency times for protein–DNA interactions can serve various roles, from promoting correct site recognition to facilitating functional processes to contributing to stable transcription activation or repression.^27,46–48^ SMARCA4’s long residency time suggests that G4 acts as a stable tether, and it is possible that this could affect the functional consequence of the interaction. It is also interesting that we observe that the SMARCA4 helicase domain is the main contributor of binding to G4s, since this domain is known to be critical for tethering SMARCA4 to chromatinized DNA.^21^ Our work provides a biophysical perspective on the SMARCA4–G4 interaction as a foundation for future investigations into how this interaction influences key genome mechanisms.

## Supplementary Material



## Notes

Oligonucleotide sequences, CD spectra, and supporting BLI data (PDF)
