# Supplementary material for: Dissecting the Binding Interactions of the Chromatin Remodeler SMARCA4 with G‑Quadruplex DNA
Source: Biochemistry. 2026 Feb 27;65(6):670–7. doi: 10.1021/acs.biochem.5c00750 (PMC13001090; doi:10.1021/acs.biochem.5c00750)
Supplement: Supplementary file 1 [file bi5c00750_si_001.pdf]

Supporting information

Dissecting the binding interactions of the chromatin remodeller SMARCA4 with G-quadruplex DNA

Sarah K. Madden[1], David Tannahill[2] and Shankar Balasubramanian[1,2,3*]

[1]Yusuf Hamied Department of Chemistry, University of Cambridge, Cambridge, CB2 1EW, UK.

[2] Cancer Research UK Cambridge Institute, Cambridge, CB2 0RE, UK.

[3]School of Clinical Medicine, University of Cambridge, CB2 0SP, UK.

[*]Correspondence to sb10031@cam.ac.uk

**Oligonucleotide**	**Sequence (5’ to 3’)**
Kit1 G4	AGGGAGGGCGCTGGGAGGAGGGTTTTT-tgbiotin
Kit1 mut	AGTGAGTGCGCTGTGAGGAGTGTTTTT-tgbiotin
Kit1 8-aza 7-deazaguanine	Biotin-AGXGAGXGCGCTGXGAGGAGXG
Kit1 ds	Same as G4 Kit1 with unlabelled reverse complement CCCTCCTCCCAGCGCCCTCCCT
Pu27 G4	TGGGGAGGGTGGGGAGGGTGGGGAAGGTTTTT-tgbiotin
Pu27 mut	TGGAGAGGATCCGGAGCGTGGAGAAGGTTTTT-tgbiotin
VEGF G4	CGGGGCGGGCCTTGGGCGGGGTTTTTT-tgbiotin
VEGF mut	CGGTGCGTGCCTTGTGCGTGGTTTTTT-tgbiotin
Kit* G4	GGCGAGGAGGGGCGTGGCCGGCTTTTT-tgbiotin
Kit* mut	TGCGAGTAGGTGCGTGTCCGGCTTTTT-tgbiotin
BCL2 G4	GGGCGCGGGAGGAATTGGGCGGGTTTTT-tgbiotin
BCL2 mut	GTGCGCGTGAGGAATTGTGCGTGTTTTT-tgbiotin
G3T	GGGTGGGTGGGTGGGTTTTT-tgbiotin
G3T2	GGGTTGGGTTGGGTTGGGTTTTT-tgbiotin

Table S1: Nucleic acid oligonucleotides used in this study

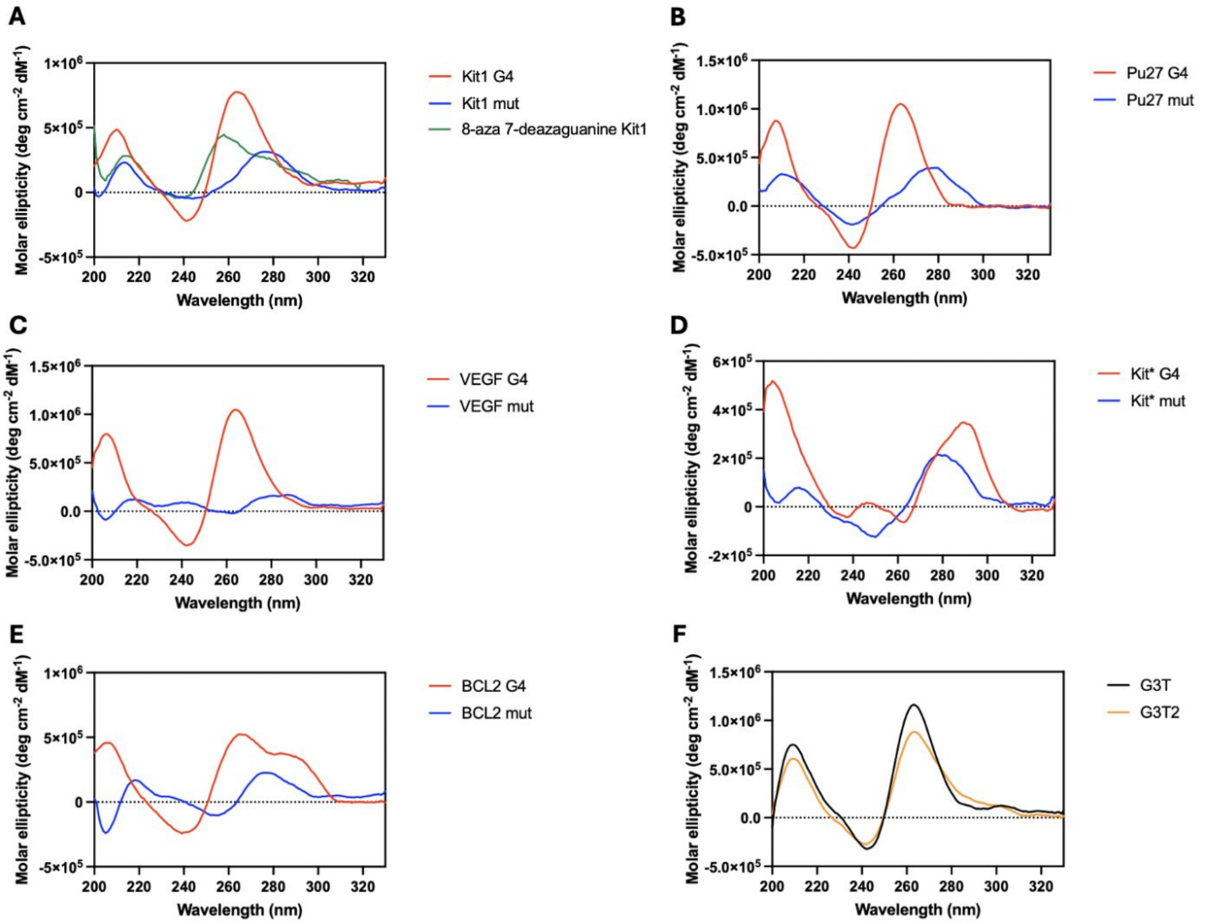

Figure S1: G4 oligonucleotides (Red) used in this study form the G-quadruplex structure as demonstrated by CD with a positive peak at 260 nm and negative peak at 245 nm for parallel G4s, a positive peak at 295 nm and a negative peak at 260 nm for anti-parallel G4s and a positive peak at 295 and 260 nm a negative peak at 245 nm for hybrid G4s.(1) Control single-stranded sequences (Blue, Green) do not for a G-quadruplex as demonstrated by CD. CD spectra of A) Kit1 G4, Kit1 mut and 8-aza 7-deazaguanine Kit1 B) Pu27 G4 and Pu27 mut, C) VEGF G4 and VEGF mut, D) Kit* G4 and Kit* mut, E) BCL2 G4 and BCL2 mut. F) In addition, two G4s with minimal loop structure G3T (Black) and G3T2 (Orange) form G-quadruplex structures as demonstrated by CD. Three readings were taken and averaged with 10 µM oligonucleotide in 10 mM Tris 100 mM KCl pH 7.4.

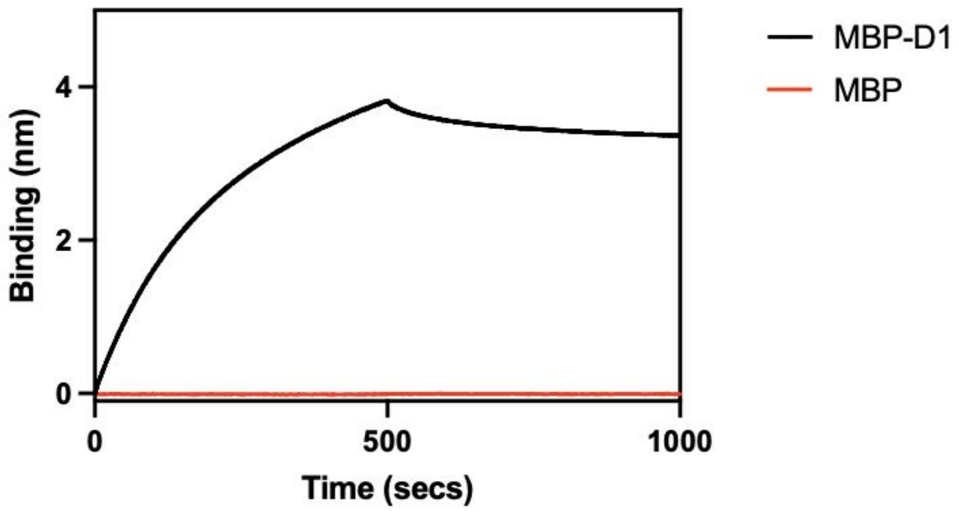

Figure S2: Comparison of binding of 500 nM MBP-D1 and 500 nM MBP to G4 Kit1 by BLI, with MBP showing no binding to G4 Kit1. Experiment carried out with 100 nM Biotinylated G4 Kit1 oligonucleotide in 50 mM Tris 200 mM KCl 5 % glycerol 1 mM DTT 1mM $MgCl_2$ 0.2 % BSA pH 7.

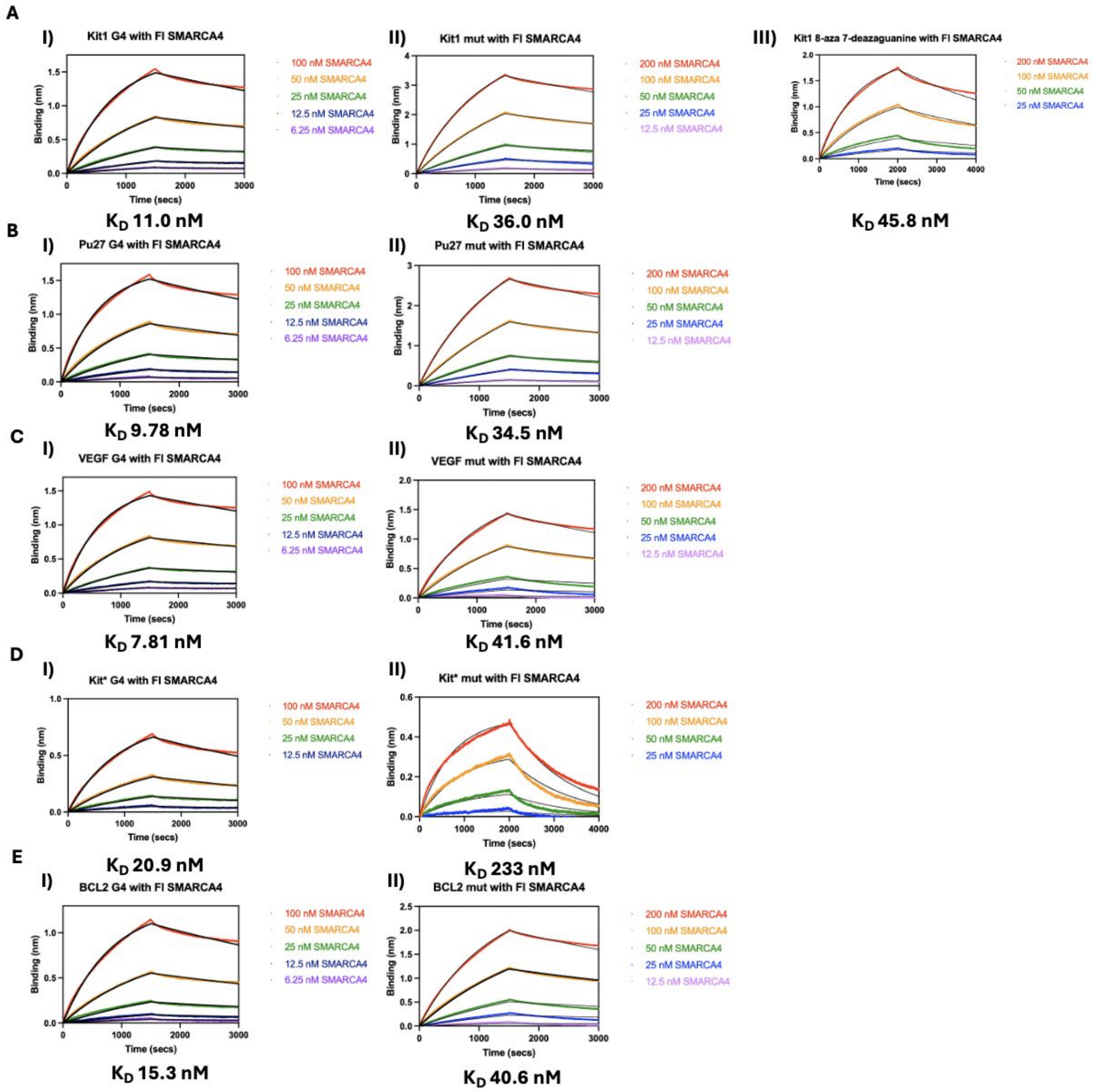

Figure S3: SMARCA4 binds tightly to a range of naturally occurring G4s with different topologies and is selective for the G4 structure. Example BLI data for the binding of all G4s and their control sequences with full-length SMARCA4 with 100 nM immobilised oligonucleotide in 25 mM TRIS 100 mM KCl 20 % glycerol 2 mM EDTA 1 mM DTT 2 % BSA pH 7.9: A) I) Kit1 G4, II) Kit1 mut and III) 8-aza 7-deazaguanine Kit1 B) I) Pu27 G4 and II) Pu27 mut C) I) VEGF G4 and II) VEGF mut D) I) Kit* G4 and II) Kit* mut E) I) BCL2 G4 and II) BCL2 mut.

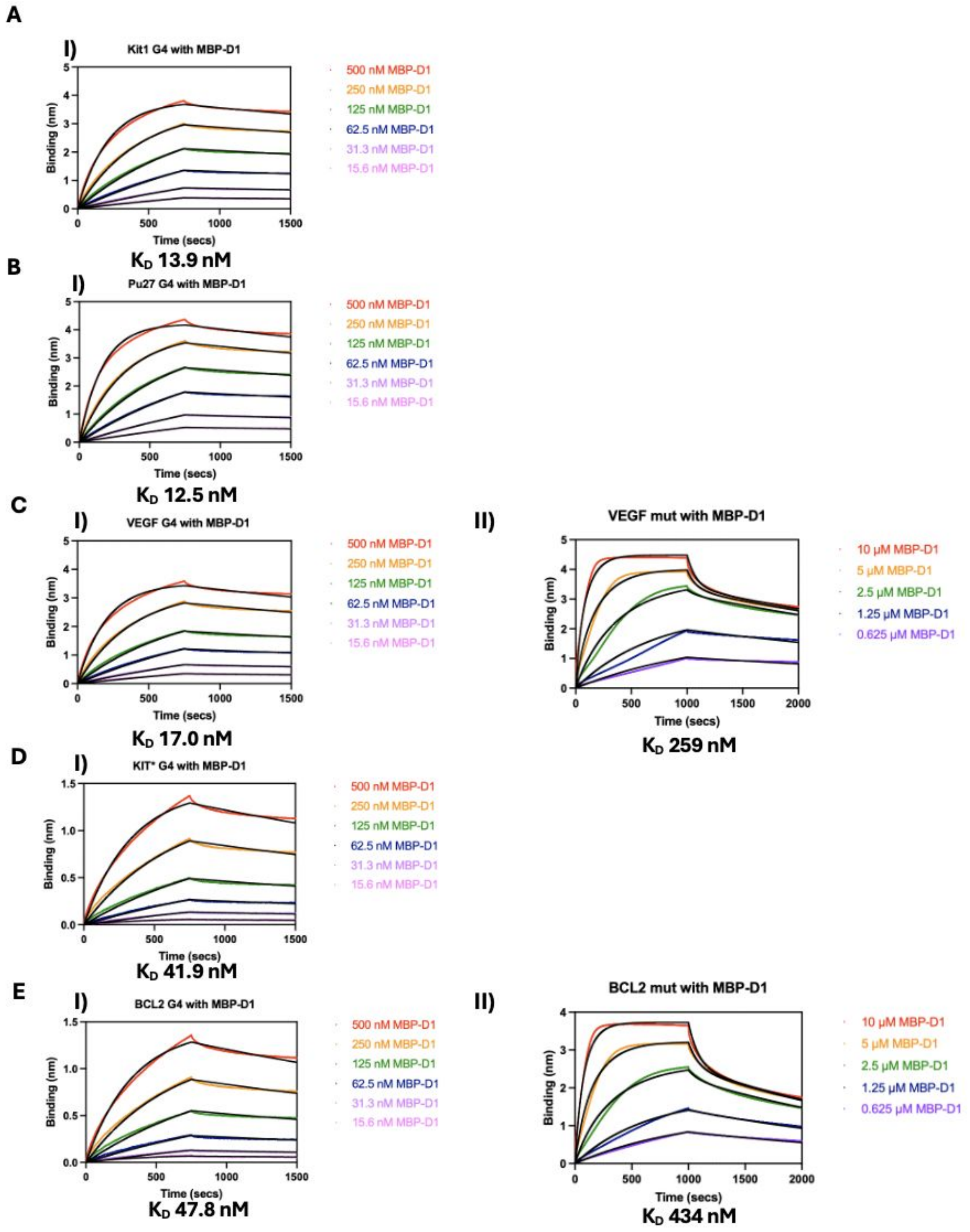

Figure S4: The SMARCA4-G4 interaction is driven by the D1 helicase domain of SMARCA4, with nanomolar affinities with a selectivity for the G4 structure being observed. Example BLI data for the binding of all G4s and their control sequences with MBP-D1 with 100 nM immobilised biotinylated oligonucleotide in 50 mM Tris 200 mM KCl 5 % glycerol 1 mM DTT 1mM $MgCl_2$ 0.2 % BSA pH 7: A) I) Kit1 G4 B) I) Pu27 G4 C) I) VEGF G4 and II) VEGF mut D) I) Kit* G4 E) I) BCL2 G4 and II) BCL2 mut.

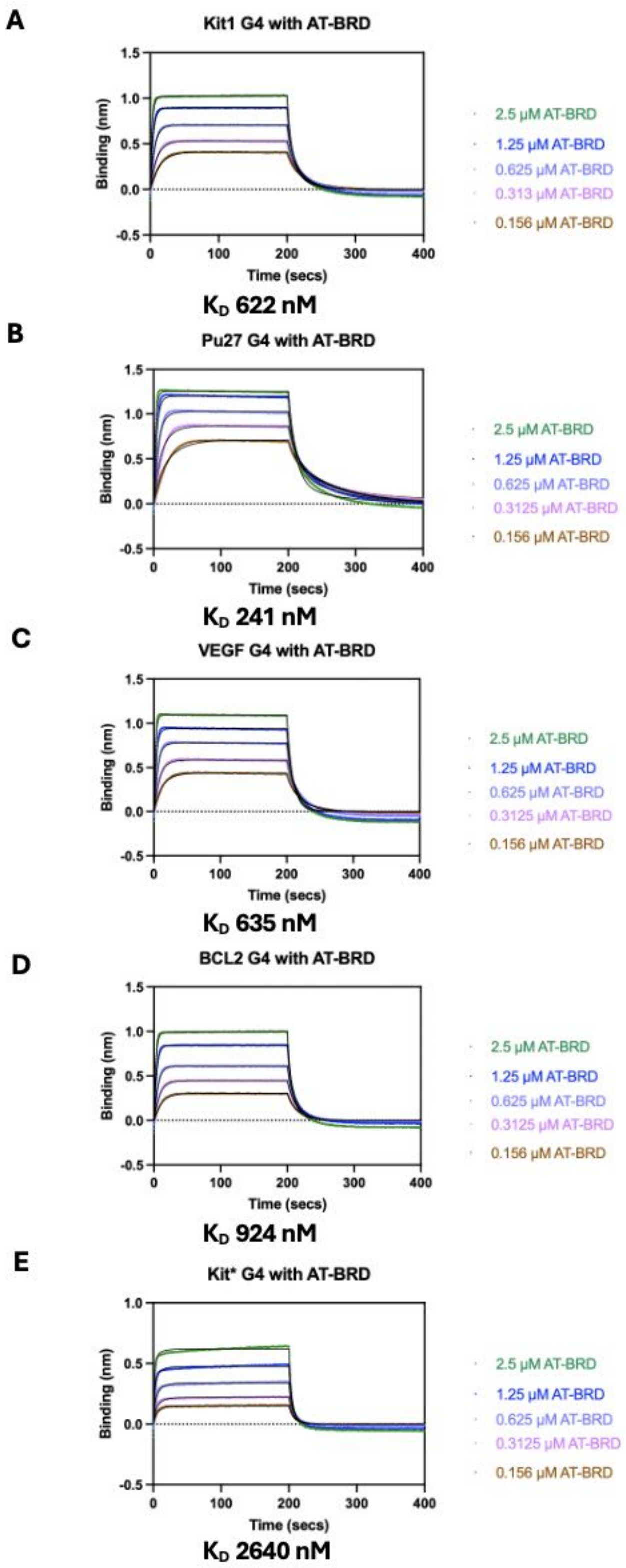

Figure S5: AT-BRD binds to G4s with micromolar affinity with a selectivity for the G4 structure being observed. Example BLI data for the binding of all G4s and their control sequences with AT-BRD with 100 nM immobilised oligonucleotide in 50 mM Potassium Phosphate 50 mM KCl 1 mM DTT 0.5 mM EDTA 0.1 % BSA pH 7: A) Kit1 G4 B) Pu27 G4 C) VEGF G4 D) BCL2 G4 E) Kit* G4.

REFERENCE

1. del Villar-Guerra, R., Trent, J. O., and Chaires, J. B. (2018) G-Quadruplex Secondary Structure Obtained from Circular Dichroism Spectroscopy. *Angewandte Chemie*. 130, 7289–7293
